# Towards quantifying the communication aspect of resilience in disaster-prone communities

**DOI:** 10.1038/s41598-024-59192-3

**Published:** 2024-04-17

**Authors:** Adaeze Okeukwu-Ogbonnaya, George Amariucai, Balasubramaniam Natarajan, Hyung Jin Kim

**Affiliations:** 1 Department of Computer Science, Kansas State University, Manhattan, KS 66502 USA; 2 Department of Electrical and Computer Engineering, Kansas State University, Manhattan, 66502 USA; 3 Landscape Architecture and Regional & Community Planning, Kansas State University, Manhattan, 66502 USA

**Keywords:** Computational science, Natural hazards

## Abstract

In this study, we investigate the communication networks of urban, suburban, and rural communities from three US Midwest counties through a stochastic model that simulates the diffusion of information over time in disaster and in normal situations. To understand information diffusion in communities, we investigate the interplay of information that individuals get from online social networks, local news, government sources, mainstream media, and print media. We utilize survey data collected from target communities and create graphs of each community to quantify node-to-node and source-to-node interactions, as well as trust patterns. Monte Carlo simulation results show the average time it takes for information to propagate to 90% of the population for each community. We conclude that rural, suburban, and urban communities have different inherent properties promoting the varied flow of information. Also, information sources affect information spread differently, causing degradation of information speed if any source becomes unavailable. Finally, we provide insights on the optimal investments to improve disaster communication based on community features and contexts.

## Introduction

Disasters can occur in any community, whether rural, suburban, or urban. However, disaster preparedness, response, and recovery differ across communities. Rural communities typically have low population density and access to essential critical services may be challenging. On the other hand, urban areas may have better access to and more robust critical infrastructures. However, since urban communities are much more heavily populated, any disaster-related disruption could have a significant impact. This contrast between urban and rural communities presents challenges in determining the optimal allocation of limited resources during disasters. Each community has to deal with its own local factors that can hamper its ability to respond to disaster. Amongst these factors, effective communication of life-saving information during a disaster is critical, and the unique characteristics of communities can affect the speed at which such information spreads, potentially impacting a community’s resilience. Our research investigates how the process of information diffusion, which is indicative of a community’s communication resilience, depends on the community’s characteristics.

In our research, “disaster” conditions refer to significant disruptions or emergencies resulting from natural disasters such as floods, hurricanes, or severe storms. These conditions can lead to widespread damage to infrastructure, displacement of populations, and interruption of essential services. However, in this study, we focus on communities that are prone to flooding and how the diffusion time during normal and disaster times differs among these communities.

In the context of flood disasters, the provided disaster times signify the duration it takes for critical information to reach various segments of the population. During these times, people are likely to receive information through multiple channels, including news broadcasts, social media updates, messages from friends and family, and official government alerts. However, the timing of when individuals receive these messages may vary depending on factors such as the availability of communication channels, individuals’ media preferences, and the effectiveness of government alert systems. Understanding the implications of disaster times on communication channels and information dissemination is indeed crucial, as it sheds light on how communities cope with and respond to emergencies. For example, during a flood, individuals might rely more on immediate sources like social media and friends for real-time updates on the situation, especially if traditional communication infrastructure like television and radio are disrupted. In contrast, on a normal day, people might primarily receive news through traditional media channels. Additionally, the types of alerts issued during a disaster, such as evacuation orders or safety advisories, would also influence people’s behaviors and the urgency with which they seek information.

Traditionally, we may define a *community* as a group of people living in close geographical proximity. Multiple studies highlight the importance of a community-based approach in enhancing disaster resilience^1–5^. However, with the recent increase in the use of digital devices and social media for communication, it is necessary to redefine the notion of *community* as people build interpersonal relationships beyond their physical neighborhoods using digital devices. People use social network platforms such as Facebook and Twitter to share information concerning disasters^6–8^. Hence, it makes sense to define the community-based social network approach to disaster resilience by augmenting face-to-face networks with other forms of social networking. Our work expands on the definition of community by including face-to-face and online social networks.

The usage of different communication mechanisms differs across communities—for instance, in communities in the United States, studies show that adults in urban and suburban communities are more likely to have access to broadband internet than their rural counterparts^9,10^. Individuals in these communities are exposed to information from diverse sources and rely on different communication methods in different ways, as various factors influence their preference for communication techniques. Our approach considers that community structure varies in different contexts, including the relationship and dependence between people and specific infrastructures, which affects the time of information diffusion. Network structure, trust, and interaction rates between people and communication infrastructures play a crucial role.

The study of resilience in complex networks is crucial for assessing the impact of failures and implementing mitigation techniques^11^. Understanding resilience is especially important for communities during natural disasters, as communication infrastructure failures can cause information disconnection. Different communities face different challenges, such as face-to-face communication difficulty when roads or bridges are inaccessible or TV and digital device failure during power outages. Over-reliance on a few methods of information diffusion creates problems when infrastructures fail. Our study looks at the impact of communication infrastructure failure on each community and the ways to improve a community’s resilience to such disruptions.

Previous studies have explored the evolution of disaster information on online social networks like Twitter and Facebook^12,13^, but little work exists for face-to-face networks or other offline communication methods. Our approach investigates the complex spread of information in disaster-affected communities by analyzing the interplay between five information sources: online social networks, local news, government sources, mainstream media, and print media. In this study, we have specific objectives as follows: First, we investigate whether there is significant variability in the information flow graph between rural, suburban, and urban communities as well as between disaster and non-disaster scenarios. Second, we investigate whether there is more information diffusion in online social networks between different community settings where spatial connectivity and infrastructure provision are different. Finally, we explore whether there is significant variability in network properties within the disaster and regular (non-disaster) information networks from rural to urban communities.

Our study finds that urban communities rely more on online social networks than rural communities for information dissemination. Furthermore, the information network properties and flow graphs of urban, suburban, and rural communities differed in normal and disaster situations, with variations in network centrality but no significant variability in the average shortest path lengths. We developed a stochastic model using survey responses to simulate information diffusion in a multilayered network of three Midwest US counties. We inferred interaction and trust patterns among people in rural, suburban, and urban communities and analyzed the effects of different sources of information on information spread. We found that community structure, the degree of sparsity, and connectedness influence the average time of diffusion of information. Moreover, the failure of one communication infrastructure affects each community differently. To overcome the need for accurate network information, we learned the distributions of the node-to-node relationships from survey responses and created synthetic graphs using these distributions.

Our contributions include the development of a stochastic model that predicts the average time of information diffusion in urban, suburban, and rural areas and districts in normal and disaster times using multiple social networks. This model integrates various factors such as community degree distribution, trust relationships, and interaction rates between individuals and communication infrastructure. Additionally, we demonstrate the independence of diffusion time from community size, even with a community graph ranging from 1000 to 25,000 nodes, assuming the same type of information is being propagated in each community in normal times or disaster times. Furthermore, we analyze information flow graphs to understand how different network structures impact diffusion time and we evaluate community resilience in scenarios of communication infrastructure failure. Finally, we develop a framework for extracting policy recommendations, enabling communities to make informed, budget-conscious investments to enhance information diffusion during disasters.

## Literature review

The independent cascade model^14,15^ and linear threshold model^15^ are the two main models used in information diffusion theory to describe the diffusion of information in a network. In the linear threshold model, every node *i* has a threshold $\tau _i$. For every edge (*i*, *j*) between *i* and its neighbor *j* there exists an influence (of *i* from *j*) weight of $w_{ij} \ge 0$ such that $\sum _{j}^{} w_{ij} <= 1$. A node *i* becomes active when the sum of the influence weights from its active neighbors surpasses $\tau _i$. By contrast, in the independent cascade model, each edge (*i*, *j*) has a certain probability in the directed graph, such that an active node *j* can influence node *i* with probability $P_{ij}$. Through this activation process, influence spreads in the network. In our work, we use a variant of these models since the propagation of information depends on two factors, trust and the rate of interactions between nodes. Hence, our work incorporates both neighbors’ influences and a trust threshold value to propagate information. Our network has rates of interaction and trust values between all the participants and their neighbors. We assume that the neighbors of an uninformed node will share information with this node during a time step. With a certain probability, informed by the nodes’ rate of interaction, an uninformed node meets an informed node during a time equal to one simulation step. During the meeting, the informed node communicates the relevant information to the uninformed node, who takes it into consideration through a mechanism that depends on the trust relationship between the two nodes, and previous exposures to the same information. When the uninformed node’s trust in the new information exceeds a certain threshold, the uninformed node becomes informed.

Past research has extensively investigated the evolution of disaster information on online social networks like Twitter and Facebook. Yoo et al.^12^ characterized diffusion rates during a disaster by examining key elements of information propagation rates on Twitter data. Fan et al.^16^ studied the influence of different users on the diffusion of disaster information on Twitter, finding that early interventions from hubs increased the speed of information propagation. Kim et al.^17^ analyzed interactions between news agencies, weather agencies, and the public on Twitter during the 2017 Storm Cindy, identifying news and weather agencies as dominant sources of information. Kim and Hastak^7^ studied the role of social media data on Facebook in information propagation during disasters, identifying specific individuals as hubs. Yang et al.^18^ investigated influential Twitter users during Hurricane Harvey, highlighting the role of objective information sharing in disaster-related follower growth. Fan et al.^13^ studied dynamic changes in network structure during information diffusion of disaster information on social networks. Dong et al.^19^ created an information flow model from Weibo data to characterize information diffusion patterns during disasters. Sharma et al.^20^ investigated challenges of integrating social network data and emergency management to maximize information diffusion of disaster information. Zhang et al.^21^ explored the potential of social media in enhancing public information and warning systems for disaster management, emphasizing its role in acquiring situational awareness and enabling communication with disaster management agencies. Zhai et al.^22^ characterized disaster tweets to understand different perspectives of shared data during information diffusion. Xu and Qiang^23^ used retweets to model information diffusion and analyze the geographic distribution of topics during information flow. Nagar et al.^24^ characterized social media response by studying how disaster news spread on Twitter and the countries where tweets emerged. Zhu et al.^25^ built a probabilistic model for retweeting during disasters, identifying factors affecting retweeting behaviors. Altay and Pal^26^ used an agent-based model to demonstrate the importance of information hubs in the speed of information during disaster response. Liu et al.^27^ developed a theoretical model for modeling information diffusion in online social networks during emergencies.

From the review of past studies, we deduce that most work on understanding information diffusion in social networks is focused on online social networks with little work on face-to-face networks or other offline communication methods. This research aims to address this gap by exploring how humans communicate using different communication mechanisms (local government, online social networks, local news, cable news, and print media) across different development contexts (urban, suburban, and rural) and identifying opportunities for enhancing communication strategies to improve disaster response and resilience. Our model incorporates a person’s relationship in their social network by including trust in neighbors and different communication mechanisms. We take this approach since research has shown that trust is vital in information diffusion and people’s decisions during disasters. Adali et al.^28^ emphasized the significance of trust in interpersonal relationships, particularly in the context of social networks, and introduced algorithmic methods to measure and quantify trust based on communication behavior, highlighting its relevance for understanding information flow dynamics in networks like Twitter. Wu et al.^29^ underscored the importance of trust in information diffusion processes within network theory, highlighting the influence of rational decisions based on trust levels in acquaintances, and explored how trust dynamics impact information propagation in a two-layer multiplex network, revealing that memory span and trustable acquaintances significantly affect information spreading dynamics. Moreover, Fridman et al.^30^ found that trust in government sources leads to accurate knowledge and application of preventive measures during COVID-19. In contrast, a negative relationship exists between trust in cable news and social networks. Additionally, an individual’s social network can affect their decisions during a disaster^31,32^. Specifically, Widener et al.^31^ used an agent-based model to show that social networks can influence evacuation decisions, while Haer et al.^32^ found that communication strategies and social networks affect whether or not people take protective actions during disasters. Our stochastic model incorporates people’s trust relationships within the community and with different communication hubs.

## Methodology

Here we discuss our survey methodology, feature extraction, graph generation, stochastic model, and model assumptions.

### Data

A total of 2,736 households were selected using a stratified cluster sampling method from urban, suburban, and rural residents within Riley County, Kansas, Buchanan County, Missouri, and Platte County, Nebraska which were recently exposed to flooding. These areas are in the high-risk Midwest watershed areas.

**Figure 1:**
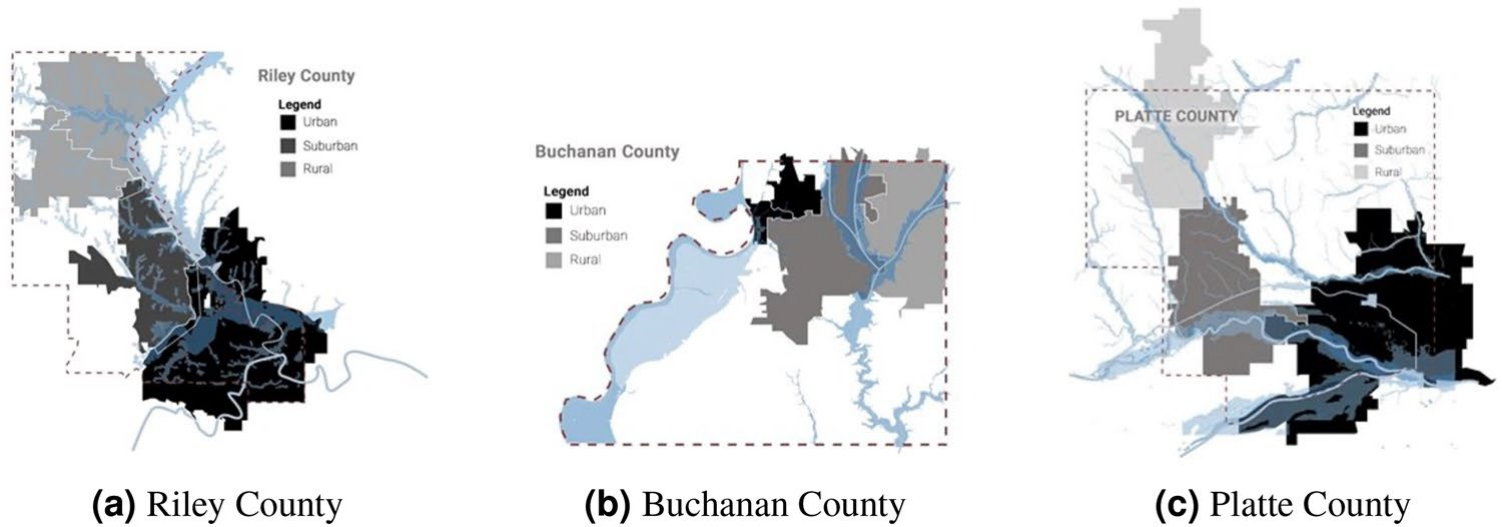
Classification of areas in county. These maps were created using Adobe Photoshop (version 22.1, Adobe Systems, https://adobe.com/products/photoshop.html)).

In our selection methodology, we considered the extent and direction of the urban-to-rural gradient within each setting, utilizing zip codes as the unit of analysis. We specifically incorporated demographic, economic, and physical characteristics to define the study areas, including population density, household income, street networks, urbanization patterns, and flood impact areas along this gradient. The resulting classification of each county into urban, suburban, and rural zones is depicted in Fig. 1. This approach allowed for a comprehensive examination of the diverse urban and rural landscapes present in our study areas.

The survey was conducted from March to April 2021 through the USPS every door direct mail (EDDM) service and 155 complete responses were obtained with a response rate of 5.6%. Before the survey was conducted, this study was approved by the Kansas State University’s Institutional Review Board. Table 1 shows the distribution of responses.

**Table 1:** Distribution of responses of communities.

Area type	Response	Districts	Response
Rural	49	Buchanan Rural	21
		Platte Rural*	5*
		Riley Rural	23
Suburban	62	Buchanan Suburban	16
		Platte Suburban	13
		Riley Suburban	33
Urban	44	Buchanan Urban*	4*
		Platte Urban	21
		Riley Urban	19

*Removed due to insufficient data.

We focus on survey responses relevant to our study of developing a network of nodes (people) with edges that describe interaction patterns and trust relationships. Although our data is limited because we do not have complete network information, we assume that survey results from random samples of nodes from different communities provide a close estimation of the interactions between community members. Since we are interested in people’s interaction and behavior in normal daily life and disaster times, our survey questions cover both situations. These responses allow us to build different information networks for disaster and normal daily life. The data includes: (i) the number of people with whom respondents interact regularly within their neighborhood, which represents the degree distribution of communities, ranging from 0 to 160; (ii) the five people respondents trust the most outside their households, from whom they prefer to obtain information during normal daily life and disaster situations—‘the frequency of interaction with them’ (a five-point Likert scale: Never =1 to Always =5)’ as well as ’the level of trust in them’ (0–10 scale); and, (iii) respondent’s preferred information source during normal and disaster times among the local government, online social networks, local news, cable news, and print media—’the frequency of weekly access to these sources (a five-point Likert scale: Never =1 to Always =5)’ and ’the level of trust in these sources’ (0–10 scale).

After the survey data cleaning, we have ten communities to explore, as shown in Table 1. We dropped two districts (Buchanan Urban and Platte Rural) due to insufficient responses but included their data when considering total urban and rural areas. To create features for each community, we convert survey responses into two sets of thirteen features—one for normal times and one for disaster times. The features are in Table 2.

**Table 2:** The features of a community.

Community features	
1. Features relating to node degree:	
a. The number of people each person interacts with daily	
2. Features relating to degree of interaction	
b. The frequency of interaction with 5 most trusted neighbors	
c. The frequency of interaction with local government	
d. The frequency of interaction with online social networks	
e. The frequency of interaction with cable news	
f. The frequency of interaction with local news	
g. The frequency of interaction with print news	
3. Features relating to degree of trust	
h. Trust value for neighbors for 5 most trusted neighbors	
i. Trust value for local government	
j. Trust value for online social networks	
k. Trust value for cable news	
l. Trust value for local news	
m. Trust value for print news	

We obtained each community’s degree sequence by using the node’s degree, which is the number of connections a node has with others^33^. We cleaned up the data by dropping one missing data point and outliers, defined as data points above 100. For the frequency of interaction with each information source and interactions with neighbors, we dropped the missing data points and encoded responses with a scale of 1–5 (Never: 1, Rarely: 2, Sometimes: 3, Frequently: 4, Very Frequently: 5). Trust values for neighbors and information sources range from 0 to 10 and we dropped missing data points for these as well.

### Graph generation

To build a stochastic model of information diffusion in each community, we need a graph representation of the community. We use the thirteen features obtained in the previous section to build graphs of the ten communities. Our assumptions to generate these graphs and assign attributes to the communities are the obtained degree distribution from the degree sequence represents the degree distribution in each community, the frequency of interaction and trust relationships indicate the strength of ties between nodes. and each person in a community can obtain information directly from five sources: cable news, online social networks, local government agencies, local news, and print media.

In reality, the populations of the communities of interest are much larger than our available data. To overcome the small sample size, we assume that the number of nodes in our graph, denoted as *n*, represents the size of each community. Our graph $G = (V, E)$ has *n* vertices and *m* edges. We generate graphs that only contain community members (without hubs) by using the kernel density estimation (KDE)^34^ to learn the degree distribution of each community. We sample and generate a degree sequence of size *n* from this distribution. We use the Markov chain Monte Carlo (MCMC) algorithm^35,36^ to sample 100 graphs from the configuration model^37^ for each community during the simulation process. We add five information source nodes (hubs) to the generated graphs—to represent thes local government, online social networks, local news, cable news, and print news—increasing the number of nodes to $n+5$. Each node can be in one of two states: with information (state 1) or without information (state 0). Before the simulation, we assume all information sources are in state 1, and all other nodes are in state 0. We build the graph using the NetworkX^38^ Python library.

Since information propagation in a network depends on tie strength^39^, we focused on understanding node interaction rates and trusts to achieve our goal of propagating information through the network. We consider two types of edge relationship attributes: interactions and trust. For interactions, we have directed edges from the sources of information to all other nodes and undirected edges between nodes. The frequency of interaction between two nodes *i* and *j* is denoted by $\lambda _{ij}$. For trust relationships, we have directed edges from nodes to sources of information and undirected edges between nodes. The degree of trust between two nodes *i* and *j* is denoted by $\theta _{ij}$.

We use kernel density estimation to learn the trust distribution from each community’s trust sequence to each source of information. Then we randomly sample *n* values from this distribution to assign trust values to edges connecting the nodes to the hubs. This results in five different trust values for each node corresponding to their ties with the five hubs. Next, we use kernel density estimation to learn the distribution of trust levels between people in a community and assign trust values to five randomly selected edges of the graph. We assign these sampled trust values to the edges representing the ties between a node and its five most trusted neighbors, and we set a constant value of 5 for the remaining edges between a node and its other neighbors.

As mentioned above, we converted the weekly interaction rate to numbers ranging from 1 to 5. We use kernel density estimation to learn the distribution for the frequency of interactions with sources of information and people in each community and sample from these distributions. For the sources of information, we get *n* samples from the learned distribution. We get $n \times 5$ random samples from the learned distribution for interactions with people. We convert these frequencies to a weekly interaction rate by assuming a typical range of days for talking with friends between [0,12] days. We assign a rate to each edge by uniformly selecting a value from a corresponding range: 0 days for frequency 0, 1–2 days for frequency 2, 3–4 days for frequency 3, 5–7 days for frequency 4, and 8–12 days for frequency 5. We convert these rates to hourly rates by dividing the number of days by $24 \times 7$. We assign the sampled rate values to only five random edges that depict an individual’s five most trusted friends and a constant rate, $\frac{5}{ 24 \times 7 }$, to the remaining edges between node *i* and its neighbors.

### Stochastic model

We are interested in estimating the average diffusion time, defined as the time that elapses between the start of an information event, and the time when 90% of individuals in the community have accepted the information (or have become *informed*). We use a stochastic model to simulate information passing within the built graphs. We start with an initial graph with *n* + 5 nodes. Initially, we assume there is a piece of information to be shared, and only the five hubs have this information. Let *X* be the number of interactions a node has with a specific neighbor—another person or a source of information—in a week, where *X* $\sim$ Poisson($\lambda$), and $\lambda$ is an edge value between the two nodes. Using this rate, we calculate the probability of 0 meetings between a node and its neighbors. Zero meetings between node *i* and a neighbor *j* mean that node *i* and *j* have not met yet, so node *i* does not learn any information from node *j*. We begin by iterating through all the nodes in the graph. For each time step, we check the state of the node. If a node has the information, we do not take any action. However, if a node does not have the information, we check its informed neighbors. We iterate through the informed neighbors of each node *i*. To determine the probability that node *i* meets node *j*, $P_0$, let the rate of interactions between *i* and *j* be $\lambda _{ij}$. We calculate ${P_0}$ as 1 minus the probability of zero meetings between node *i* and node *j*, that is, ${P_0} = 1 - Poisson(X=0,\lambda _{ij}) = 1 - e^{\lambda _{ij}}$.

We draw once from a Bernoulli distribution with parameter $P_0$. If the outcome is 1, we update the uninformed node’s total trust in the new information. To update this total trust, we apply a discount factor to the trust value between node *i* and *j*. The discount factor accounts for the number of times node *j* passed the same information to node *i* before. Previous interactions with node *j*, as well as with other nodes, are discounted by a forgetting factor that accounts for the length of time that passed since those interactions. We calculate the trust as follows:

1 $$\begin{aligned} T_c = \sum _{j=1}^N\sum _{k=0}^{M_{ij}} \theta _{ij}d^{k}f^{t_c - t_k } \end{aligned}$$

$T_c$ is the total trust in the new information, *N* is the number of neighbors of *i* with a state of 1, $M_{ij}$ is the number of meetings between *i* and *j*, $\theta _{ij}$ is the trust between node *i* and *j*, *d* is the discount factor and *f* is the forgetting factor, $t_c$ is current time step and $t_k$ is the time of meeting number *k* between *i* and *j*. Node *i* becomes *informed* when $T_c$ exceeds a threshold $\Theta$. In this experiment, we assign a discount factor $d=0.5$, a forgetting factor $f=1.3$, and a trust threshold value $\Theta =30$. Next, we conduct a sensitivity analysis for these parameters, and the results are in the Results section. The simulation ends when 90% of the population receives the information. We repeat the simulation 1000 times for each of the 100 graphs and take an average of the diffusion time. See Algorithm 1 in the Appendix for the pseudocode.

### Network metrics

We chose several key network metrics for our analysis, each providing insights into the structure and dynamics of community networks and their significance in understanding information propagation within communities. We computed these network metrics using algorithms implemented in NetworkX^38^ and Gephi^41^. We discuss the metrics in the following paragraphs.

The number of edges in a network reflects the connections between nodes, indicating overall connectivity and the density of relationships within the community^44^. More edges suggest a denser network with increased communication channels, fostering efficient information exchange and collaboration among members. Similarly, the number of triangles signifies closed loops formed by interconnected nodes, revealing the level of clustering within the network^33,42^. A high level of clustering can facilitate local information sharing and collaboration among closely-knit groups.

The average clustering coefficient quantifies how closely nodes cluster in the network, indicating the prevalence of tightly interconnected groups^33^. A higher coefficient suggests a greater tendency for clustering, facilitating efficient information diffusion and collaboration. Meanwhile, the average path length measures the average shortest distance between all node pairs^43^,signifying information spread efficiency. A shorter path length enables faster communication and coordination, crucial for community disaster resilience as critical information spreads quickly among individuals, enhancing overall resilience.

The average degree signifies the average connections per node^41^, reflecting network connectivity and influence. A higher average degree suggests stronger communication networks, aiding information dissemination during emergencies. On the other hand,the average weighted degree offers a nuanced measure, considering both connection number and strength^41^. It highlights nodes with stronger relationships, facilitating effective information exchange and collaboration within the community network.

### Diffusion time prediction with Gaussian process regression

Communities need to identify target areas for the improvement of communication resilience during disasters. We can approach this problem as a regression task where community features help to predict diffusion time. However, with only ten communities, we need more data points. We generate synthetic features using the original ten communities to increase the number of data points, focusing on features during disaster events. We generate 1000 data points using the algorithms explained in the following subsection. After generating the synthetic graphs and features, we simulate them using Monte Carlo simulations and obtain the mean and standard deviation of diffusion time from 1000 simulation runs. Afterward, we use a Gaussian Process Regression Model to predict the diffusion time for the ten original communities using features obtained through synthetic data generation. We split the dataset into train and test by a ratio of 80:20. We use the sum of DotProduct and WhiteKernel as the kernel of the regression model. We discuss the results in the Results section.

We utilize two algorithms for generating synthetic graphs that closely resemble the original data. Both employ kernel density estimation to learn the degree distribution from the initial survey data. Algorithm 2 applies slight perturbations subsequently to the mean and standard deviation of the degree sequence to introduce variability. Algorithm 3 adjusts histogram bin heights. Sampling from these distributions generates a degree sequence of size $d=1000$. Further details and implementation steps can be found in the Appendix. Furthermore, we generate synthetic trust and interaction data to label graph edges, aiming to mimic the original community features closely. Initially, we adjust the relative frequencies of ordinal variables by ±0.1 and renormalize them. Trust and interaction values are then generated based on these adjustments. For detailed implementation, see Algorithm 4 in the Appendix.

### Ethical standards in human participant research

In this study, we adhered to the necessary ethical considerations in research involving human participants. This study was approved by the Kansas State University’s Institutional Review Board. We ensured strict compliance with relevant guidelines and regulations and we confirm that informed consent was duly obtained from all participants.

## Results

We discuss the results and provide answers to the questions in the Introduction using the data and experimental results.

### Network properties of information networks

The structure of community networks serves as a crucial determinant in understanding the dynamics of different communities, particularly during normal and disaster situations. Throughout our analysis of the network structures, we observe variations in network properties across different community types and between normal and disaster situations. These variations highlight the nuanced dynamics of community networks and their implications for information diffusion and community resilience.

Our analysis encompasses several key network properties, as outlined in Table 3. Across the various communities, we discern the following trends:

**Table 3:** Network properties of information network.

Community (5005 nodes)	Normal Times						Disaster times					
	Number of Edges	Number of Triangles	Average Clustering Coefficient	Average Path Lengths	Average Degree	Average Weighted Degree	Number of Edges	Number of Triangles	Average Clustering Coefficient	Average Path Lengths	Average Degree	Average Weighted Degree
Buchanan Rural	45751	87723	0.277	1.997	18.282	0.46	46548	95353	0.294	1.996	18.601	0.468
Buchanan Suburban	25322	19388	0.262	2.033	10.119	0.218	25941	20789	0.282	2.011	10.366	0.252
Platte Suburban	39272	62845	0.345	2.002	15.693	0.431	38402	62158	0.372	2	15.345	0.421
Platte Urban	38185	50716	0.239	2.027	15.259	0.373	38587	57622	0.263	2.006	15.345	0.372
Riley Rural	46361	83500	0.215	2.006	18.526	0.485	48307	94272	0.231	1.999	15.419	0.511
Riley Suburban	35351	47340	0.325	2.005	14.126	0.341	36044	53147	0.355	1.997	19.303	0.372
Riley Urban	30538	32017	0.279	2.031	12.203	0.293	30664	34468	0.312	2.002	14.403	0.314
Rural	45736	83108	0.254	2.002	18.276	0.469	47336	95551	0.269	1.998	12.253	0.493
Suburban	33577	42120	0.312	2.01	13.417	0.324	33680	44299	0.340	1.999	18.915	0.347
Urban	34511	42839	0.268	2.023	13.791	0.335	34542	46396	0.296	2.003	13.803	0.348

In Buchanan Rural, during disaster times, there is a slight increase in both the number of edges and triangles compared to normal times. This suggests that there might be more interactions or connections among nodes in the network during disasters, leading to higher clustering coefficient. Similarly, Buchanan Suburban experiences an increase in the number of edges and triangles during disasters, alongside a more noticeable rise in the average clustering coefficient. This indicates a denser network structure and potentially stronger local connections among nodes.

Platte Suburban shows a relatively stable number of edges between normal and disaster times, with a slight decrease in the number of triangles during disasters. Nonetheless, the average clustering coefficient increases, implying a more clustered network structure during disasters. Platte Urban witnesses a slight increase in both the number of edges and triangles during disaster times. Notably, the average clustering coefficient sees a significant increase, suggesting a more tightly knit network with stronger local connections among nodes.

Riley Rural experiences an increase in both the number of edges and triangles during disasters, indicating a denser network structure. However, the average clustering coefficient remains relatively stable, suggesting that the increase in connections may not necessarily result in stronger local clustering. Similarly, Riley Suburban sees an increase in both the number of edges and triangles during disasters. However, the average clustering coefficient sees a significant increase, indicating a more clustered network structure with stronger local connections. In Riley Urban, there is a slight increase in both the number of edges and triangles during disaster times. Notably, the average clustering coefficient witnesses a notable increase, suggesting a more tightly clustered network with stronger local connections among nodes.

In rural areas, during disaster times, there is an increase in both the number of edges and triangles, indicating a denser network structure. However, the increase in the average clustering coefficient is relatively small, suggesting a moderate increase in local clustering. Similarly, in suburban areas, there is an increase in both the number of edges and triangles during disaster times. However, the average clustering coefficient sees a more significant increase, indicating a denser and more clustered network structure during disasters. Finally, in urban areas, there is a slight increase in both the number of edges and triangles during disaster times. The increase in the average clustering coefficient is notable, suggesting a more tightly knit network with stronger local connections among nodes during disasters.

In general, average path lengths remain relatively stable across communities during both normal and disaster times, indicating consistent information transmission efficiency regardless of conditions. However, differences in community structures are evident across area types in our dataset. Variations in average path lengths suggest differing efficiency levels in information transmission within each area type. The number of edges varies, indicating denser networks in specific regions, while significant differences in the number of triangles suggest unique clustering patterns in each area type. Additionally, variations in average clustering coefficient and average degree reflect disparities in network cohesion and node connectivity specific to different areas. Similarly, variations in average weighted degree highlight diverse connection strengths inherent to specific regions.

### Information diffusion in normal and disaster times

We measure a community’s communication resilience to disasters by the time it takes for information to diffuse to 90% of the population. Our study aims to answer whether there is a significant difference in the information flow graph between rural, suburban, and urban communities and between disaster and non-disaster scenarios. During the simulation, we obtain the diffusion times and their standard deviations for all communities under study. Table 4 displays the results for different area types and districts, which show that information diffuses at varying speeds across all communities and diffuses faster in disaster situations than in normal daily life. Interestingly, rural communities have a faster diffusion rate than urban communities, even in disaster scenarios. These findings suggest significant variability in the information flow graph between communities.

**Table 4:** Diffusion times reaching to 90% of population.

Community	Normal time		Disaster time	
	Mean (hours)	Std	Mean (hours)	Std
Buchanan Rural	22.366	0.12	20.447	0.111
Buchanan Suburban	38.341	0.279	32.016	0.208
Platte Suburban	22.091	0.059	22.069	0.057
Platte Urban	27.091	0.183	26.767	0.163
Riley Rural	27.784	0.21	24.360	0.158
Riley Suburban	25.784	0.118	22.977	0.069
Riley Urban	30.038	0.192	25.843	0.155
Rural	24.395	0.152	21.881	0.091
Suburban	27.469	0.145	24.600	0.115
Urban	27.966	0.169	25.091	0.135

**Figure 2:**
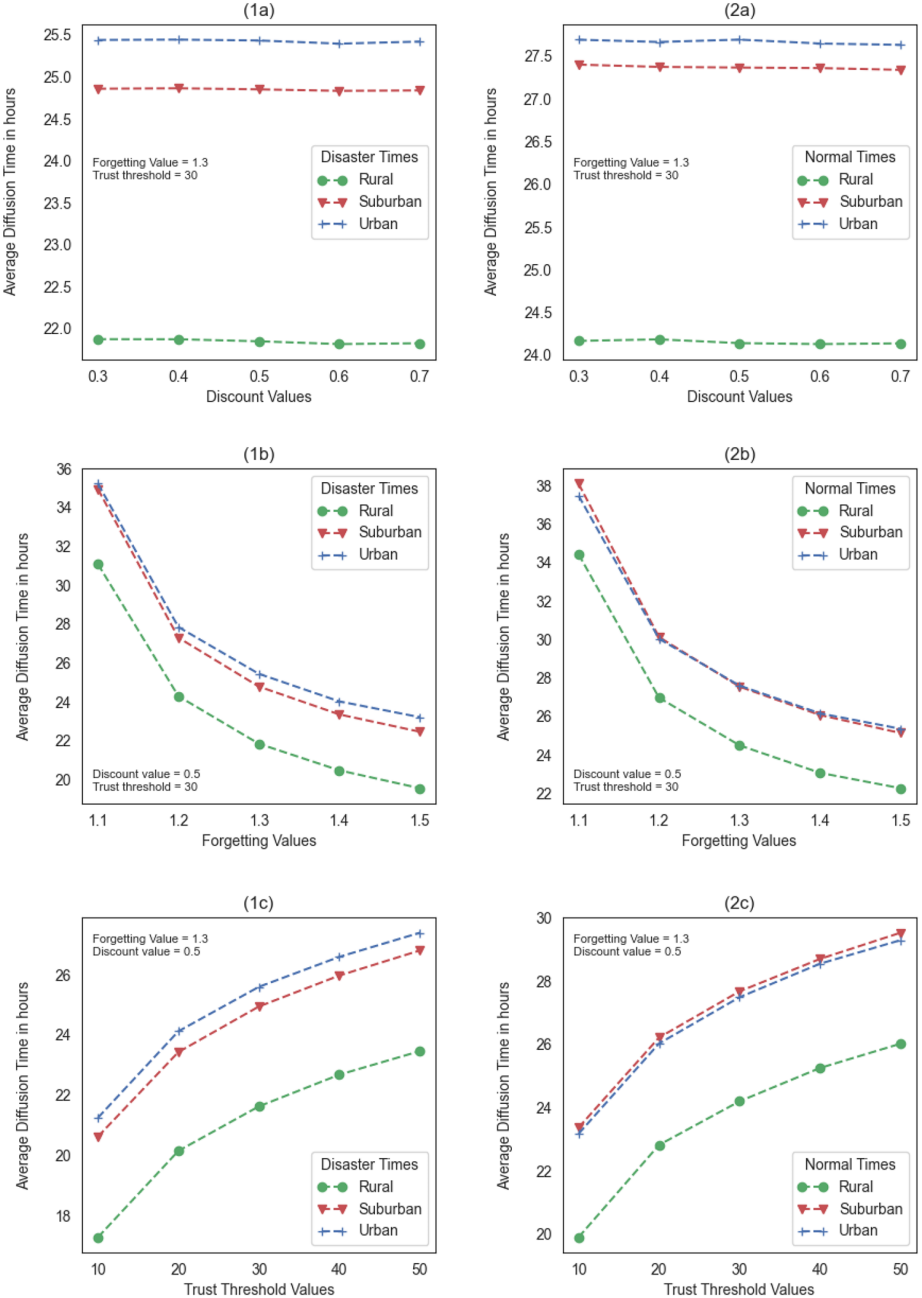
Sensitivity analysis of the average diffusion time during disaster (1a, 1b, 1c) and normal (2a, 2b, 2c) times.

We conduct a sensitivity analysis to examine how sensitive our results are to changes in the discount, forgetting, and trust threshold values. The analysis results are presented in Fig. 2. We observe that the trends of the diffusion time plots are consistent for the discount, forgetting, and trust threshold values in both disaster and normal scenarios. Rural communities have a significantly different diffusion time than urban and suburban communities. Interestingly, for certain forgetting factor values in normal times, we observed similar diffusion times in urban and suburban communities. Similarly, for the plot of the diffusion times using different trust thresholds in normal scenarios, urban and suburban communities are close.

### Resilience to information hub failure

We recognize that infrastructures are vulnerable, especially during disasters. Therefore, it is crucial to incorporate redundancies in infrastructure networks. For communication networks such as the one we are studying, it is essential to have multiple communication paths when some of the infrastructure fails. To investigate the impact of infrastructure failure on communities, we adjust our model by dropping one of the five hubs and observing the resulting diffusion time. We examine rural, suburban, and urban communities response to the loss of a particular infrastructure since communities rely on specific communication infrastructure.

**Figure 3:**
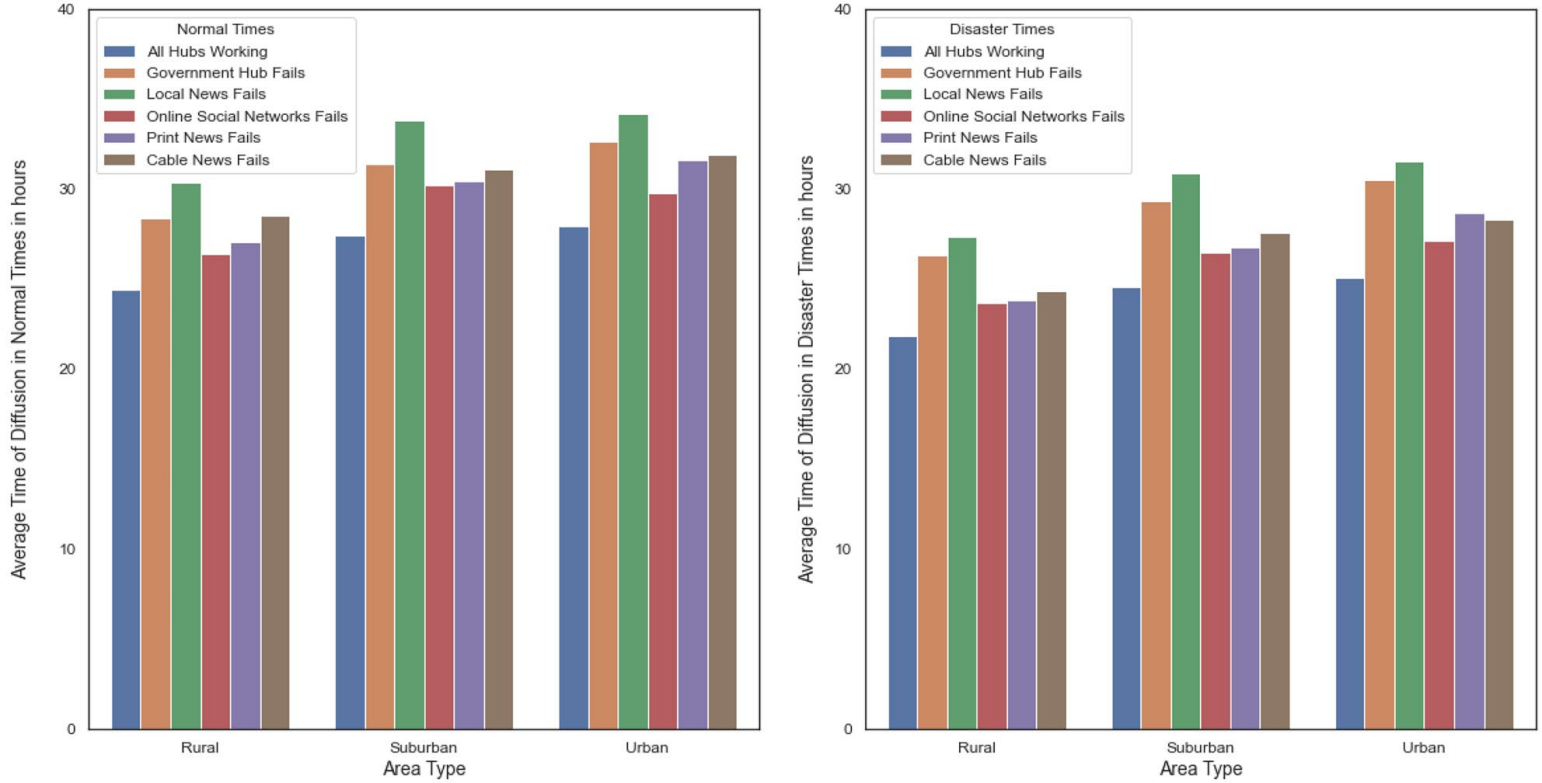
Average diffusion times when one hub fails against all hubs working in disaster and normal times.

We compare these simulation results to our initial results when all hubs participate in the diffusion process. The results are in Fig. 3, which indicates that the diffusion time increases when a source of information fails, both in normal and disaster times. In normal times, the failure of online social networks seems to have the most significant impact on the time of information diffusion across all area types, with the most pronounced effect in urban areas, suggesting heavy reliance on social media in these areas. Government hub failure appears to have a moderate impact, again with urban areas showing the highest increase in diffusion time, indicative of their reliance on government communication channels. Local news and print news failures show a relatively less impact on the diffusion time, with the least effect seen in rural areas, potentially due to alternative information sources being utilized. Cable news failure seems to have the least impact among all hubs across all area types.

During disaster times, the failure of each communication hub generally results in a greater increase in diffusion time compared to normal times. The failure of online social networks continues to have the most substantial impact, particularly in suburban areas, suggesting that these areas might be most affected by social media outages in emergencies. Government hub failure shows a significant increase in diffusion time in rural areas compared to urban and suburban, indicating that rural areas may be more dependent on government communication during disasters. Local news failure appears to affect suburban areas the most, while cable news failure has the least impact, similar to the pattern observed during normal times.

Overall, the reliance on communication hubs varies by area type and is exacerbated during disaster times. Urban areas show a high dependence on online social networks and government communication, suburban areas are significantly reliant on online social networks, and rural areas show increased reliance on government hubs during disasters.

The greater spatial disconnection in urban areas compared to rural communities implies that urban dwellers may rely more on online social networks (OSN) than rural residents. To investigate this, we compare the diffusion time when the OSN fails while other hubs are active to the time when all hubs are functioning. Table 5 shows that the mean difference in diffusion time between all hubs working and when the online social network hub fails in urban communities during a disaster is higher than in rural or suburban communities. For disaster scenarios, absolute time in hours matters the most, more than the percentage change in time. Hence, we interpret this result to mean that a high difference indicates a high reliance on OSN. With this perspective we observe that, in a disaster scenario, urban communities rely on OSN more than other communities. However, in normal times, suburban communities are most affected when OSN fails. These findings suggest that urban communities tend to rely more on OSN compared to rural and suburban areas, with suburban communities being most affected during normal times when the OSN hub fails.

**Table 5:** Reliance of urban and rural communities on online social networks.

Community	Normal (All hubs)		Normal (OSN Fails)		Mean Difference	Disaster (All hubs)		Disaster (OSN Fails)		Mean Difference
	Mean (hours)	Std	Mean (hours)	Std		Mean (hours)	Std	Mean (hours)	Std	
Rural	24.395	0.152	26.392	0.161	1.997	21.881	0.091	23.698	0.122	1.817
Suburban	27.469	0.145	30.231	0.189	2.762	24.600	0.115	26.472	0.127	1.871
Urban	27.966	0.169	29.809	0.202	1.843	25.091	0.135	27.143	0.149	2.052

### Impact of population size on time of diffusion

We tested the impact of the number of nodes on information diffusion times to ensure that our simulation results were not biased. We varied the number of nodes to include 1000, 5000, 10,000, 15,000, 20,000, and 25,000 nodes and kept the graph features constant for each community. Our findings showed no significant differences (p-value=0.1) in the diffusion times across different population sizes of similar communities, as presented in Table 6.

**Table 6:** Comparison using different number of nodes in network.

Size	Platte Suburban (Disaster and No Government Hub)		Rural Disaster		Riley Urban Normal	
	Mean (hours)	Std	Mean (hours)	Std	Mean (hours)	Std
1000	25.409	0.293	22.038	0.222	29.771	0.407
5000	24.860	0.242	21.881	0.091	30.038	0.192
10000	24.901	0.059	22.015	0.033	29.723	0.13
15000	24.913	0.107	21.963	0.027	29.835	0.101
25000	25.000	0.003	21.986	0.01	29.635	0.135

### Gaussian process regression model results

The Gaussian Process Regression model provides us with a reliable estimate of the time it takes for information to spread, along with the uncertainty surrounding this estimate. We obtain the average diffusion time for test data with corresponding standard deviations. The mean absolute percentage error (MAPE) for the regression is 4.574%, which suggests a reasonable forecasting performance for the regression model. Using our model, we make predictions for the ten communities as shown in Table 7. We observe that our predictions closely align with actual diffusion times.

**Table 7:** Gaussian process model predictions for the 10 community groups.

Community	Average time of diffusion (hours)	Standard deviation	Predicted time of diffusion (hours)	Standard deviation
Buchanan Rural	20.660	1.393	20.447	0.111
Buchanan Suburban	33.007	1.402	32.016	0.208
Platte Suburban	22.995	1.395	22.069	0.057
Platte Urban	27.334	1.394	26.767	0.163
Riley Rural	25.222	1.393	24.360	0.158
Riley Suburban	24.866	1.398	22.977	0.069
Riley Urban	26.625	1.395	25.843	0.155
Rural	21.623	1.394	21.881	0.091
Suburban	25.444	1.405	24.600	0.115
Urban	25.744	1.396	25.091	0.135

For good response planning, we need to understand the speed at which information disseminates during a disaster. Accurate predictions enable community leaders and emergency responders to make informed decisions, prioritizing areas that require immediate attention. The use of original community data ensures the applicability of our predictions to real-world scenarios. This direct connection to community data enhances the value of our forecasts for decision-makers, providing insights grounded in actual community dynamics. Additionally, our predictions can be utilized to simulate various disaster scenarios, offering communities the opportunity to assess and refine their response strategies. By comprehensively understanding how different scenarios influence communication and response times, communities can bolster their preparedness efforts.

#### Budget allocation methodology using gradients

A community’s unique characteristics may be crucial to improve its disaster resiliency strategies. Using the resilience metrics of diffusion time, we provide a budget allocation method for a community interested in improving the time of diffusion during a disaster. The average diffusion time *T* is a multi-variable function $\bar{f}(p_1, p_2,\ldots , p_n)$, learned through the Gaussian Regression model, with parameter vector $p=[p_1, p_2,\ldots , p_n]^T$, which is the feature vector of each community.

We use a gradient approach to develop the budget allocation method. We calculate the partial derivative, $\triangledown {\bar{f}(p)}$, as below:

2 $$\begin{aligned} \triangledown {\bar{f}(p)} = \begin{bmatrix} \frac{\partial {f(p)}}{\partial {p_1}} ~ \frac{\partial {f(p)}}{\partial {p_2}} ~ \ldots ~ \frac{\partial {f(p)}}{\partial {p_n}} \end{bmatrix}^T = \begin{bmatrix} \frac{\bar{f}(p_1+\delta p_1,p_2,\ldots ,p_n) - \bar{f}(p_1,p_2,\ldots ,p_n)}{\delta p_1}~ \ldots ~ \frac{\bar{f}(p_1,p_2,\ldots ,p_n+\delta p_n) - \bar{f}(p_1,p_2,\ldots ,p_n)}{\delta p_n} \end{bmatrix}^T \end{aligned}$$

We used $\delta p_i=1$ for each feature $p_i$ in the vector space, except in one case, where the feature’s magnitude was high and we used a value of 10. We calculate the gradient at the values of the feature vectors specific to each community. To evaluate $\bar{f}$ in the modified values of the feature vector (i.e., $\bar{f}(p_1+\delta p_1,p_2,\ldots ,p_n),~ \bar{f}(p_1,p_2+\delta p_2,\ldots ,p_n), \ldots \bar{f}(p_1,p_2,\ldots ,p_n+\delta p_n)$) we use the developed Gaussian Regression model.

See Appendix, Table 1 for the gradient results. From our results, we find that the five most critical features, in order of importance, are the Median Frequency of Interaction with Cable News, the Median Trust for Online Social Networks, the Median Frequency of Interaction with the Local Government, the Mean of the Degree distribution of the Community Network, and the Median Trust for People. These listed features correspond to the highest (in absolute value) gradient components for each community and could be where the communities might want to focus.

To guide community investments based on the calculated gradient, we propose the following simple strategy. Let the vector $\Delta =[\Delta _1, \Delta _2, \ldots \Delta _n]^T$ denote the changes in the community feature vector achievable by the investment of one monetary unit.—e.g., $\Delta _i$ can be the change in $p_i$ achieved by investing one dollar in changing $p_i$ (increasing it or decreasing it, depending on whether the gradient component $\frac{\partial {f(p)}}{\partial {p_i}}$ is negative or positive, respectively). Then the gradient with respect to investments, and which, after normalization (so that the sum of the components becomes 1) should inform how one dollar is spent, is given by $\triangledown {\bar{f}(p)} * \Delta$, where $*$ denotes the Hadamard product. One single dollar should then be split amongst the community feature components as indicated by the vector$\frac{1}{|\triangledown {\bar{f}(p)} * \Delta |_1}\triangledown {\bar{f}(p)} * \Delta$, where $|\cdot |_1$ denotes the $L_1$ norm.

## Discussion

The diffusion times during normal and disaster scenarios, as presented in Table 4, highlight variations among different community types. In regular circumstances, diffusion times range from approximately 22.09 to 38.34 hours, indicating gradual information spread. During disasters, diffusion times decrease across all community types, suggesting faster information dissemination, albeit with some variability based on community context. These findings emphasize the intricate relationship between community attributes and information diffusion dynamics, underscoring the need for tailored communication strategies.

The Gaussian Process Regression (GPR) model results provide reliable estimates of diffusion time, with a low mean absolute percentage error (MAPE) of 4.574%, indicating reasonable forecasting performance. By predicting diffusion times for different communities, valuable insights for response planning are provided, enabling leaders and responders to prioritize areas needing immediate attention. Moreover, the model’s accuracy is demonstrated through close alignment with observed diffusion times.

The gradient analysis identifies critical factors influencing diffusion time across communities. Key influencers include interaction frequency with cable news and online social networks, trust in the local government and people, and network properties. These findings highlight the importance of social dynamics and communication channels in shaping community resilience during disasters. For instance, communities with higher interactions with local government may exhibit faster information diffusion, as residents are more likely to heed official warnings and instructions. Similarly, frequent interactions with cable news and online social networks can facilitate the rapid dissemination of critical information, enabling residents to stay informed and take appropriate actions during emergencies. Communities can utilize this information to prioritize investments or interventions targeting specific features to enhance communication resilience. By allocating resources based on gradient analysis, communities can optimize preparedness efforts, strengthening overall resilience.

Analyzing the relationship between network properties and diffusion time reveals valuable insights for disaster resilience strategies. Rural communities generally exhibit smaller diffusion times compared to suburban and urban counterparts, indicating potentially more efficient information dissemination processes during disasters. Certain network features, such as degree distribution mean, interaction frequency with cable news, and trust in online social networks, play crucial roles in shaping diffusion dynamics across different community types. Rural areas often display negative gradient values for these features, suggesting that increasing interaction frequency or trust levels in specific communication channels could expedite information spread. Conversely, suburban and urban communities show mixed gradient values, implying a more nuanced relationship between network properties and diffusion time. These findings stress the importance of tailoring communication strategies to community-specific characteristics. By leveraging insights from diffusion time analysis and gradient results, stakeholders can prioritize investments in communication infrastructure and information exchange channels to mitigate the impact of disasters on vulnerable communities. Accurate modeling and analysis are crucial for informing effective disaster response and resilience strategies, empowering communities to enhance their resilience and minimize the impact of disasters on their populations.

## Conclusion

This study highlights the need for tailored communication strategies because of these differences in communication patterns among different communities. We further emphasize that certain features are more important for each community, and investments in social programs and infrastructure should focus on improving interactions with cable news and local government and trust for online social networks. Also, we find that urban communities rely more on online social networks than rural communities for information dissemination. Moreover, the failure of one communication infrastructure affects each community differently, while the time of diffusion is independent of the size of the community.

Our study provides valuable insights for community partners and policymakers to develop effective disaster communication strategies and improve community resilience. However, it has some limitations. Firstly, the study only focuses on one type of disaster event within three US Midwestern regions, which may limit the generalizability of the findings. Future studies could explore the influence of community features in different disaster contexts and geographical locations to enhance the generalizability of the results. Secondly, this study relies on self-reported data, which may be subject to bias and inaccuracies. Future studies could use more objective measures to collect data, such as social media activity or mobile phone usage patterns. Thirdly, there was a low response rate for the survey during the COVID-19 pandemic, which might violate statistical validity based on the lack of sample size. Additionally, the absence of data on shared contacts between individuals limits the depth of our analysis. Future research could aim to address this limitation by exploring novel methodologies for collecting such data. Also, we note that another limitation is that the single-step gradient approach, while useful for developing a budget allocation method for policy recommendations, may oversimplify the complexity of policy implementation, potentially neglecting essential factors critical for effective execution. Finally, future studies could delve deeper into the mechanisms to understand better how specific community features influence information diffusion during a disaster. Despite these limitations, this study’s findings offer valuable insights into how to improve communication strategies for disaster resilience in communities.

### Supplementary Information

Supplementary Information.

## Data Availability

The datasets analyzed during the current study are available from the corresponding author upon reasonable request.

## Code availability

All codes are available on this GitHub repository^40^.
