# Supplementary material for: Towards quantifying the communication aspect of resilience in disaster-prone communities
Source: Sci Rep. 2024 Apr 17;14:8837. doi: 10.1038/s41598-024-59192-3 (PMC11024194; doi:10.1038/s41598-024-59192-3)
Supplement: Supplementary file 1 — Supplementary Information. [file 41598_2024_59192_MOESM1_ESM.pdf]

Towards Quantifying the Communication Aspect of Resilience in Disaster-Prone Communities

Adaeze Okeukwu-Ogbonnaya[1,*], George Amariucai[1], Balasubramaniam Natarajan[2], and Hyung Jin Kim[3]

[1]Kansas State University, Department of Computer Science, Manhattan, Kansas, 66502, USA
[2]Kansas State University, Department of Electrical and Computer Engineering, Manhattan, 66502, USA
[3]Kansas State University, Landscape Architecture and Regional & Community Planning, Manhattan, 66502, USA
[*]adaeze@ksu.edu

Appendix

Stochastic Model

In this section, Algorithm 1 presents the pseudocode for the stochastic model. We are interested in estimating the average diffusion time, defined as the time that elapses between the start of an information event, and the time when 90% of individuals in the community have accepted the information (or have become *informed*). We use a stochastic model to simulate information passing within the built graphs. We start with an initial graph with $n + 5$ nodes. Initially, we assume there is a piece of information to be shared, and only the five hubs have this information. Let X be the number of interactions a node has with a specific neighbor – another person or a source of information – in a week, where $X \sim \text{Poisson}(\lambda)$, and λ is an edge value between the two nodes. Using this rate, we calculate the probability of 0 meetings between a node and its neighbors. Zero meetings between node i and a neighbor j mean that node i and j have not met yet, so node i does not learn any information from node j. We begin by iterating through all the nodes in the graph. For each time step, we check the state of the node. If a node has the information, we do not take any action. However, if a node does not have the information, we check its informed neighbors. We iterate through the informed neighbors of each node i. To determine the probability that node i meets node j, P_0, let the rate of interactions between i and j be λ_{ij}. We calculate P_0 as 1 minus the probability of zero meetings between node i and node j, that is,

$$P_0 = 1 - Poisson(X = 0, \lambda_{ij}) = 1 - e^{\lambda_{ij}}. \quad (1)$$

We draw once from a Bernoulli distribution with parameter P_0. If the outcome is 1, we update the uninformed node's total trust in the new information. To update this total trust, we apply a discount factor to the trust value between node i and j. The discount factor accounts for the number of times node j passed the same information to node i before. Previous interactions with node j, as well as with other nodes, are discounted by a forgetting factor that accounts for the length of time that passed since those interactions. We calculate the trust as follows:

$$T_c = \sum_{j=1}^{N} \sum_{k=0}^{M_{ij}} \theta_{ij} d^k f^{t_c - t_k} \quad (2)$$

T_c is the total trust in the new information, N is the number of neighbors of i with a state of 1, M_{ij} is the number of meetings between i and j, θ_{ij} is the trust between node i and j, d is the discount factor and f is the forgetting factor, t_c is current time step and t_k is the time of meeting number k between i and j. Node i becomes *informed* when T_c exceeds a threshold Θ.

Graph Generation Algorithms

For graph generation, we use two methods to generate synthetic graphs that closely resemble the original. In the first graph generation method, Algorithm 2, we first use kernel density estimation to learn the degree distribution of the degree sequence of initial survey data and sample from it to generate a degree sequence of size $d = 1000$. We shift the mean and standard deviation of the degree sequence of the initial survey data by small amounts, randomly sampled from intervals centered around zero as shown in Algorithm 2. In the second method, as shown in Algorithm 3, we change the heights of the bins of the histograms of the degree distribution. We again use kernel density estimation to learn the degree distribution of the degree sequence of

the initial survey data. By sampling from this distribution, we generate a degree sequence of size $d = 1000$. We then create a histogram of the distribution and add or subtract 0.1 from the weighted frequency of each bin to get a new frequency for each bin. We normalize the new weighted frequencies based on the sum of the original frequency of each bin in the histogram and generate points from a uniform distribution between the original bin widths. The number of points generated from each bin equaled the calculated normalized frequencies. Finally, we perform some cleanup to ensure the length of the generated data is equal to the size needed and to round up to zero if any data point is less than zero.

Algorithm 1 The Monte Carlo Simulation Algorithm

```
Require: Graph G, exposedNodes, time
initialize populationPercent = 90% of nodes in Graph
initialize time = 0
while len(exposedNodes) < populationPercent do
    d = 0.5 {discount value}
    f = 1.3 {forgetting value}
    Θ = 30 {trust threshold value}
    for i =1 to N nodes in G do
        if state of i = 0 then
            Get all the neighbors of node i, neighborNodes
            newTrust = 0
            for j in neighborNodes do
                if state of j = 1 then
                    Get zeroProbability
                    probabilityInfection = 1 - zeroProbability
                    if probabilityInfection > Uniform[0,1] then
                        if key j is in the meetings dictionary between i and j then
                            Update M by 1 {M is number of meetings of i and j}
                            Update array timeStep with current time
                            Get the θ between node j and i {refers to trust value}

                            initialize trustInStep = 0
                            for m = 0 to M do
                                Get t
                                trustInStep += d^m × f^(time−t)
                            end for
                            newTrust += ( trustInStep × θ)
                        else
                            initialize M, time, trustInStep = 0, 0, 0
                            Get the θ between node j and i
                            Update M by 1
                            Get the M {last meet number of i and j}
                            initialize t = 0
                            for m = 0 in M do
                                trustInStep += (d^m) × f^(time−t)
                            end for
                            newTrust += ( trustInStep × θ)
                        end if
                    end if
                end if
            end for
        end if
        if newTrust > Θ then
            Update State of i = 0
        else
            State of i = 0
        end if
        if State of i == 1 then
            Add node i to exposedNodes
        end if
    end for
    time += 1
end while
timeDiffusion.append(time)
return timeDiffusion
```

Algorithm 2 Graph Generation 1

```
Require: Original data d, Length L
newData = KDE(l,d) {Kernel Density Estimation(KDE) requires the graph length to generate}
oldMean = Mean of d
oldStd = Standard deviation of d
meanChange = oldMean × 0.1
stdChange = oldStd × 0.05
meanChange = Random select one of [-meanChange,meanChange]
stdChange = Random select one of [stdChange, stdChange^−1]
for all i in newData do
    i = (i + meanChange) × stdChange
    Convert i to integer and if the value is less than 0, update to 0
    newData.append(i)
end for
return newData
```

Generating Trust and Interaction Values

We discuss the algorithm for generating trust and interaction values as shown in pseudocode Algorithm 4. Since, we need to get synthetic trust and frequency of interaction data to label the edges of the synthetic graphs. We want the artificial trust and interaction data to be close to the original features of the ten communities. We first get the frequencies of each of the ordinal variables. Next, we change the sizes of the relative frequencies by adding or subtracting 0.1 from each of them. Then we renormalize the new relative frequencies and generate the new trust and interaction values according to these values. Then, the remaining code is for cleanup to ensure the length of data generated is equal to the expected length.

Algorithm 3 Graph Generation 2

```
Require: Original data d, Length L
newData = KDE(l,d) {Kernel Density Estimation(KDE) requires the graph length to generate}
n,bins = Histogram(newData) {array n is the number of counts in each bin} {array bins are the edges of the bins}
binWidth = difference between two successive bins
Get array weightfreq, the weighted frequency of each count in n
Get oldSum which is sum of elements of n
change = 0.1
Initialize empty array, newFreqList
for all i and j in bins and weightfreq do
    if j ≠ 0 then
        newFreqBin = j + UniformRV[-change × i, change × i]
    else
        newFreqBin = j + UniformRV[-change,change]
    end ifnewFreqList.append(newFreqBin)
end for
Initialize empty array, newData
for all a and b in newFreqList and bins do
    numVal = (a/(sum(newFreqList))) × 100
    newFreq = (numVal / 100) × oldSum
    gen = Generate UniformRV[b, b + binwidth] of size newFreq
    newData.append(gen)
end for
{Comment: Cleanup code to ensure data length consistency}
if length(newData) < L then
    deficit = L - length of newData
    min,max = minimum(newData), maximum(newData)
    r = DiscreteUniformRV[min,max] of length deficit
    newData.append(r)
else
    k = absolute(L - length of newData)
    Repeat newdata.pop() k number of times
end if
Change the element to 0 if the element in newData < 0
return newData
```

Algorithm 4 Generation of Trust and Interaction values

```
Require: 0riginal data d
Let array n contain the frequency of all datapoints in d
Let array keys contain the set of unique points in d
Get array relativeFreq, the relative frequency of each count in n
Get oldSum which is sum of elements of n
change = 0.1
Initialize an empty array newFreqList
for all i in relativeFreq do
    if f ≠ 0 then
        newFreq = i + UniformRV[-change × b, change × b]
    else
        newFreq = i + UniformRV[-change,change]
        newFreqList.append(newFreq)
    end if
    for all k and f in keys and newFreqList do
        numVal = (f / sum(newFreqList)) × 100
        newFreq = (numVal / 100) × oldSum
        gen = create an array with k of size f
        newData.append(gen)
    end for
end for{Comment: Cleanup code to ensure length consistency}
if length(newData) < L then
    deficit = L - length of newData
    min,max = minimum(newData), maximum(newData)
    r = DiscreteUniformRV[min,max] of length deficit
    newData.append(r)
else
    k = absolute(L - length of newData)
    Repeat newdata.pop() k number of times
end if
return newData
```

Gradient Results for the Budget Allocation Method

We outline a budget allocation method for communities aiming to enhance information diffusion time during disasters.

The average diffusion time (T) is determined by various factors, represented by a feature vector ($\mathbf{p}$), learned through a Gaussian Regression model. To develop the budget allocation method, we utilize a gradient-based approach.The gradient, which indicates the rate of change of diffusion time with respect to each feature in the vector, is calculated by comparing the average diffusion time at slightly modified feature values. Specifically, we increment each feature by a small amount (δ) and observe the resulting change in diffusion time. For most features, we set δ to 1, but for features with high magnitudes, we use a larger value, typically 10. This allows us to capture the impact of each feature on diffusion time accurately.

Using the developed Gaussian Regression model, we evaluate the average diffusion time at these modified feature values to compute the gradient. This approach guides the allocation of resources by highlighting which features have the most significant influence on improving information diffusion time during disasters.

We provide details on the Gradient Results here in Table 1.

Table 1. Table of Gradient Results

Features	Buchanan Rural	Buchanan Suburban	Platte Suburban	Platte Urban	Riley Rural	Riley Suburban	Riley Urban	Rural	Suburban	Urban
Mean of Degree Distribution	-0.986	-0.021	-0.273	-0.633	-0.337	0.689	-0.417	-1.458	-0.355	-0.546
Variance of Degree Distribution	0.436	0.121	1.149	0.789	1.085	2.111	1.005	-0.036	1.067	0.876
Skewness of Degree Distribution	3.712	0.449	4.425	4.065	4.361	5.387	4.281	3.241	4.343	4.152
Kurtosis of Degree Distribution	-0.256	0.052	0.457	0.097	0.393	1.420	0.313	-0.727	0.375	0.184
Mode of Interaction with People	0.130	0.091	0.843	0.484	0.779	1.806	0.699	-0.341	0.761	0.570
Median Interaction with People	-3.526	-0.275	-2.813	-3.173	-2.877	-1.851	-2.957	-3.998	-2.895	-3.086
Mode of Trust with People	0.982	0.176	1.695	1.335	1.631	2.658	1.551	0.511	1.613	1.422
Median Trust with People	-0.662	0.012	0.051	-0.308	-0.013	1.014	-0.093	-1.133	-0.031	-0.222
Mode of Interaction with Local Government	0.624	0.140	1.337	0.977	1.273	2.299	1.193	0.153	1.255	1.064
Median Interaction with Local Government	-2.045	-0.127	-1.331	-1.691	-1.396	-0.369	-1.475	-2.516	-1.413	-1.605
Mode of Interaction with Cable News	0.138	0.091	0.851	0.491	0.787	1.813	0.707	-0.334	0.769	0.577
Median Interaction with Cable News	-2.615	-0.184	-1.902	-2.262	-1.966	-0.939	-2.046	-3.086	-1.984	-2.175
Mode of Interaction with Online Social Network	-0.480	0.030	0.233	-0.127	0.169	1.195	0.089	-0.952	0.151	-0.040
Median Interaction with Online Social Network	0.508	0.129	1.221	0.861	1.157	2.184	1.077	0.037	1.139	0.948
Mode of Interaction with Local News	-0.517	0.026	0.196	-0.164	0.132	1.159	0.052	-0.988	0.114	-0.077
Median Interaction with Local News	1.911	0.269	2.624	2.264	2.560	3.587	2.480	1.440	2.542	2.351
Mode of Interaction with Print News	-0.263	0.051	0.450	0.090	0.386	1.412	0.306	-0.735	0.368	0.177
Median Interaction with Print News	0.596	0.137	1.309	0.949	1.245	2.272	1.165	0.125	1.227	1.036
Mode of Trust with Local Government	1.546	0.232	2.259	1.899	2.195	3.221	2.115	1.075	2.177	1.986
Median Trust with Local Government	0.925	0.170	1.638	1.278	1.574	2.601	1.494	0.454	1.556	1.365
Mode of Trust with Cable News	0.381	0.116	1.094	0.735	1.030	2.057	0.950	-0.090	1.012	0.821
Median Trust with Cable News	-2.087	-0.131	-1.374	-1.733	-1.438	-0.411	-1.518	-2.558	-1.456	-1.647
Mode of Trust with Online Social Network	0.268	0.105	0.981	0.621	0.917	1.943	0.837	-0.204	0.899	0.707
Median Trust with Online Social Network	-0.828	-0.005	-0.115	-0.474	-0.179	0.848	-0.259	-1.299	-0.197	-0.388
Mode of Trust with Local News	0.535	0.131	1.248	0.889	1.184	2.211	1.104	0.064	1.166	0.975
Median Trust with Local News	-1.707	-0.093	-0.994	-1.354	-1.058	-0.032	-1.138	-2.179	-1.076	-1.268
Mode of Trust with Print News	0.062	0.084	0.775	0.415	0.711	1.737	0.631	-0.410	0.693	0.502
Median Trust with Print News	2.638	0.341	3.351	2.991	3.286	4.313	3.207	2.166	3.269	3.077
